# Physicochemical Modeling of Electrochemical Impedance in Solid-State Supercapacitors

**DOI:** 10.3390/ma16031232

**Published:** 2023-01-31

**Authors:** Davood Peyrow Hedayati, Gita Singh, Michael Kucher, Tony D. Keene, Robert Böhm

**Affiliations:** 1 Faculty of Engineering, Leipzig University of Applied Sciences, 04277 Leipzig, Germany; 2 School of Chemistry, University College Dublin, Belfield, 4 Dublin, Ireland

**Keywords:** electrochemical impedance spectroscopy (EIS), equivalent circuit (EC), impedance modeling, solid-state supercapacitor (SSC)

## Abstract

Solid-state supercapacitors (SSCs) consist of porous carbon electrodes and gel-polymer electrolytes and are used in novel energy storage applications. The current study aims to simulate the impedance of SSCs using a clearly defined equivalent circuit (EC) model with the ultimate goal of improving their performance. To this end, a conventional mathematical and a physicochemical model were adapted. The impedance was measured by electrochemical impedance spectroscopy (EIS). An EC consisting of electrical elements was introduced for each modeling approach. The mathematical model was purely based on a best-fit method and utilized an EC with intuitive elements. In contrast, the physicochemical model was motivated by advanced theories and allowed meaningful associations with properties at the electrode, the electrolyte, and their interface. The physicochemical model showed a higher approximation ability (relative error of 3.7%) due to the interface impedance integration in a more complex circuit design. However, this model required more modeling and optimization effort. Moreover, the fitted parameters differed from the analytically calculated ones due to uncertainties in the SSC’s microscale configuration, which need further investigations. Nevertheless, the results show that the proposed physicochemical model is promising in simulating EIS data of SSCs with the additional advantage of utilizing well-reasoned property-based EC elements.

## 1. Introduction

Supercapacitors are used in a wide range of energy storage applications, such as portable electronic devices [1,2] and electric vehicles [3,4,5], thanks to their high power density, expanded cycle life, and fast charging speed. In an electric double-layer capacitor (EDLC), the electrode is made of porous carbon materials with high specific surface area which leads to a high capacitance [6]. Various carbon materials, such as activated carbon and carbon nanotubes (CNTs) [7], are used as electrode materials in EDLCs [8]. Activated carbon is the most widely used electrode material for EDLCs due to the availability of the raw materials, mature preparation methods, low cost, and nontoxicity [9]. Recently, agricultural waste has been used as a precursor material to produce activated carbon electrodes. These carbon electrodes are economical, sustainable [10], and offer a uniform porous structure [11]. Such structures often exhibit aligned channels and are ideal for fast mass transport [12], which lead to high ionic/electronic conductivity [13].

Liquid electrolytes, commonly used in conventional supercapacitors, offer good electrode/electrolyte interfacial properties and have high ionic conductivities [14]. However, they suffer from certain drawbacks for structural applications, such as hazardous material leakage [15]. Therefore, more attention has recently been given to developing gel polymer electrolytes as substitutes for liquid electrolytes due to their inherent ability to infiltrate into the pores of the electrodes while possessing other benefits in handling and packaging [16,17,18,19,20,21,22]. In other words, solid-state supercapacitors (SSCs) with gel polymer electrolytes work on a similar mechanism as their conventional counterparts but use less structural space for the same power level [23]. SSCs can potentially serve in various key applications, such as miniaturized electronics [24], wearable electronic textiles [25], microrobots [26], implantable medical devices, and sensors [27].

Nevertheless, the full realization of the SSCs’ potential is currently impeded due to the low ionic mobility and poor electrode/gel electrolyte interface [28]. These limitations impose increased internal resistance and reduced charge storage properties [29]. Therefore, gel-polymer electrolytes have a relatively high interfacial impedance [30]. This means that charges accumulate or deplete at the interface during the charging and discharging process and form an electrical double layer (EDL), commonly referred to as the space charge layer [31]. Unfortunately, the space charge layer and its impact on the charging behavior are not entirely understood [32,33].

To deepen the understanding and improve the performance of SSCs in future use cases, electrochemical spectroscopy methods such as electrochemical impedance spectroscopy (EIS) should be used. The EIS technique is a powerful electrochemical spectroscopy method that uses an alternating current (AC) signal applied to the working electrode in order to determine the corresponding cell response [34]. Steady-state polarization measurements at a constant current is also used in some studies; however, impedance analysis with an AC signal provides more information that is not available in steady-state measurements [35]. Equivalent circuit (EC) models are introduced as an effective method to interpret the EIS data. Partial elements in an EC model can be defined quantitatively and used to formulate the total cell impedance by fitting the experimental test results with the estimated impedance spectroscopy.

In general, EIS data can be modeled via two approaches: (1) a mathematical modeling approach and (2) a physicochemical modeling approach. Mathematical modeling is a top-down approach in which experimental impedances are explained using mathematical functions or equivalent ECs. These models can achieve an agreeable fit between the calculated and experimental impedances while keeping the number of parameters to a minimum. In contrast, the physicochemical modeling approach is a bottom-up method where measured impedances are linked with process parameters, such as concentrations and diffusion coefficients.

Most of the EC models used in the study of supercapacitors are based on the mathematical approach (see, for example, [36,37,38,39]). However, in the current study, mathematical and physicochemical modeling approaches were individually applied to simulate the EIS response of an SSC, manufactured from biologically based carbon electrodes and gel-polymer electrolyte. The mathematical model relied on a best-fit approach in which conventional fitting software was used to select the EC to model the electrochemical impedance data. On the other hand, a physicochemical EC model was constructed using advanced physical models. The approximation ability of both models was investigated using the EIS data of the SSC. To the authors’ knowledge, the current study is the first systematic physicochemical EC modeling attempt in solid-state supercapacitors.

## 2. Experimental Investigations

### 2.1. Preparation of Biocarbon and Free-Standing bc−GP Electrodes

The biomass precursor material (here, garlic peel, 1 g) was washed several times with water and dried for 12 h at 60 °C. The garlic peels were then placed in a 23 mL autoclave bomb reaction vessel and chemically treated with 5% aqueous MgSO_4_ (Acros Organics, Geel, Belgium) solution (10 mL). Here, MgSO_4_ was used as an activating agent to enlarge existing pores and allow the formation of new pores by opening the inaccessible pores, thereby preventing ash formation [40]. The reaction was hydrothermally treated for 12 h at 230 °C. The obtained mixture was dried for another 12 h in a hot-air oven at 60 °C. The dried powder mixture was calcined for 2 h in a tubular furnace at 700 °C and heated up with 10 °C/min ramp under an inert argon atmosphere. The as-obtained black carbon derived from biomass was used as an active electrode material, biologically based carbon (BioCarbon), hereinafter referred to as bc−GP. The bc−GP powder materials were made into free-standing electrodes using a dry-film process for electrochemical characterization. To fabricate these electrodes, a mixture of 0.2 g of Super P Carbon Black, 99.9+% (Alfa Aesar, Haverhill, MA, USA), and 1.8 g of bc−GP were ground manually using a mortar and pestle. This mixture was homogenized with 200 μL of PTFE solution (60 wt.% dispersion in water, Sigma-Aldrich, St. Louis, MO, USA) in a mortar and pestle containing 60 mL of ethanol. The above mixture was blended for 30 to 45 min until a soft dough-like mass was acquired. The dough was rolled into a sheet and dried for at least 12 h at 80 °C to obtain a 0.17 mm thick sheet, which was later cut into 10 mm disc-shaped electrodes.

### 2.2. Preparation of Gel-Polymer Electrolyte

**Figure 1:**
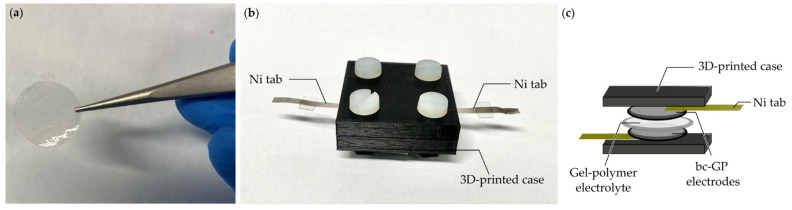
(**a**) Disc-shaped PVA-KOH-KCl gel-polymer electrolyte; (**b**) original setup; (**c**) schematic of the fabricated SSC setup.

The alkaline gel-polymer electrolyte was prepared with slight modifications, as described by Yang et al. [41]. The gel-polymer electrolyte was developed using the solution casting method and polyvinyl alcohol (PVA) with a molecular weight of approximately 50,000 $g{\cdot \mathrm{mol}}^{-1}$ (Thermo Scientific, Waltham, MA, USA). Furthermore, an aqueous 6 M KOH (Fisher Chemical, Hampton, NH, USA) solution was mixed for 4 h at 80 °C with a weight ratio of 1:2. After complete dissolution, a 0.1 M KCl was further added and blended for another 30 min. The mixture was stirred vigorously for a few seconds to attain a homogeneous viscous appearance. This mixture was poured into a clean and dry Petri dish, and a dropper with suction bulb was used to remove all air bubbles. The Petri dish was then transferred to an oven at 80 °C for 12 h to remove the excessive water. The as-prepared PVA-KOH-KCl gel-polymer electrolyte was cut into a 12 mm disc-shaped electrolyte with a thickness of 0.36 mm (Figure 1a).

### 2.3. Fabrication of SSC Cell

The SSC cell was fabricated in a symmetrical configuration, as shown in Figure 1b,c. The 12 mm width disc-shaped PVA-KOH-KCl gel-polymer electrolyte was sandwiched between two 10 mm bc−GP disc-shaped electrodes (Figure 1c). The gel-polymer electrolyte acts both as an electrolyte and a separator. The sandwiched structure was placed in a thick additive-manufactured polymer block and was screwed tightly by Teflon screws (Figure 1b). The SSC cell was allowed to rest for 10 to 12 h before conducting the electrochemical analysis.

### 2.4. Microstructure, Surface Morphology, and Electrical Characterization

**Figure 2:**
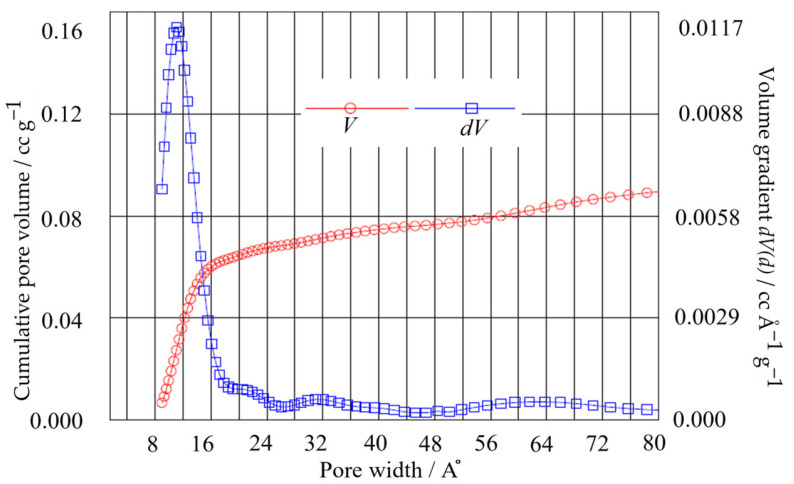
BET pore volume distribution of bc−GP from desorption isotherms using the DFT method.

The surface morphology of the bc−GP was investigated using scanning electron microscopy (SEM, Mira3 LMH, Tescan, Dortmund, Germany) at 30 kV. In this study, the surface area of the electrode was obtained using the Brunauer–Emmett–Teller (BET) surface area analysis. Additionally, the density functional theory (DFT) method was employed to determine the pore volume and pore width of the electrode (see Figure 2 and Table 1). Using the results, two key parameters (i.e., porosity fraction (χ) and pore radius ($r_{p}$)) were obtained, which were then utilized in calculating the internal resistance of the porous electrode in Equation (7). Here, an automated gas sorption acquisition and reduction DFT instrument (Autosorb iQ, Quantachrome Instruments, Boynton Beach, FL, USA) was employed using nitrogen gas at a bath temperature of 77.4 K. Figure 2 presents the BET data obtained according to the DFT method with a pore radius of ~11 A°.

Furthermore, the electrochemical properties were measured using an electrochemical workstation system (VSP-3e, BioLogic, Seyssinet-Pariset, France). EIS measurement of the SSC system was conducted in the frequency range from ω = 1 mHz to ω = 100 kHz at an amplitude of 50 mV. Cyclic voltammogram (CV) analysis was also carried out (see Appendix A.1) to investigate the power limitations of the SSC [42]. All of the electrochemical measurements were carried out in a two-electrode configuration.

## 3. Impedance Modeling

**Figure 3:**
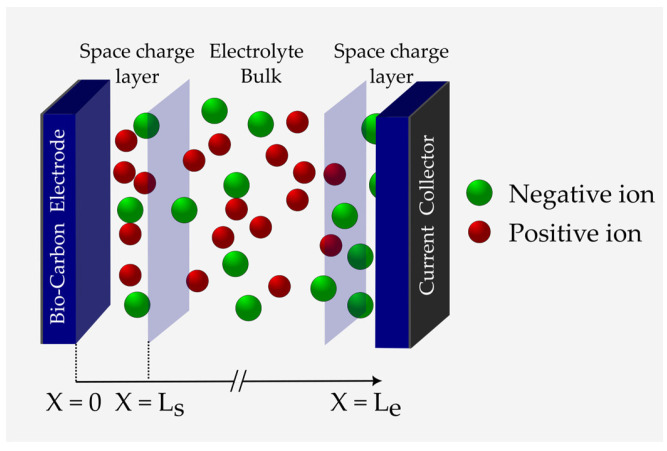
Schematic of an SSC and the corresponding dimensions $L_{s}$ of the space charge layers and $L_{e}$ of the total electrolyte length (graphic inspired by [44]).

An SSC cell generally comprises two porous planar electrodes attached to metal current collectors and contains a gel-polymer electrolyte in the middle. From a microscopic view, when a perturbation potential is introduced to an SSC, an atomistic-scale space charge layer is formed in the electrode/electrolyte interfaces next to the bulk electrolyte (Figure 3) [43].

For an arbitrary perturbation signal, where the applied potential perturbation is sinusoidal, the impedance can be defined as the ratio of potential and current phasors, i.e., $Z\left(j\omega \right)=\tilde{E}\left(j\omega \right)/\tilde{I}\left(j\omega \right)$, where  Z is the complex impedance ($Z=Z^{\prime }+jZ^{\prime \prime }$), ω is the angular frequency, and $j=\sqrt{-1}$ is the imaginary unit [35]. EIS uses the signal as a function of frequency at a constant potential and is conventionally depicted in impedance plots, called complex plane plots, or more commonly Nyquist plots. These plots present the real impedance as a function of the imaginary impedance [45]. Nyquist complex curves contain key information, usually used for calibrating mechanisms and determining the kinetics of processes, resistances, and capacitances [35].

The present study followed two independent approaches to model the impedance response. First, a conventional mathematical model was obtained and used to approximate the measured impedances. Secondly, an advanced physicochemical model was derived and applied to simulate the EIS data. Finally, the best-fit parameters were determined and compared to analytically driven values by fitting these models to the experimental data.

### 3.1. Mathematical Approach to Modeling

**Figure 4:**
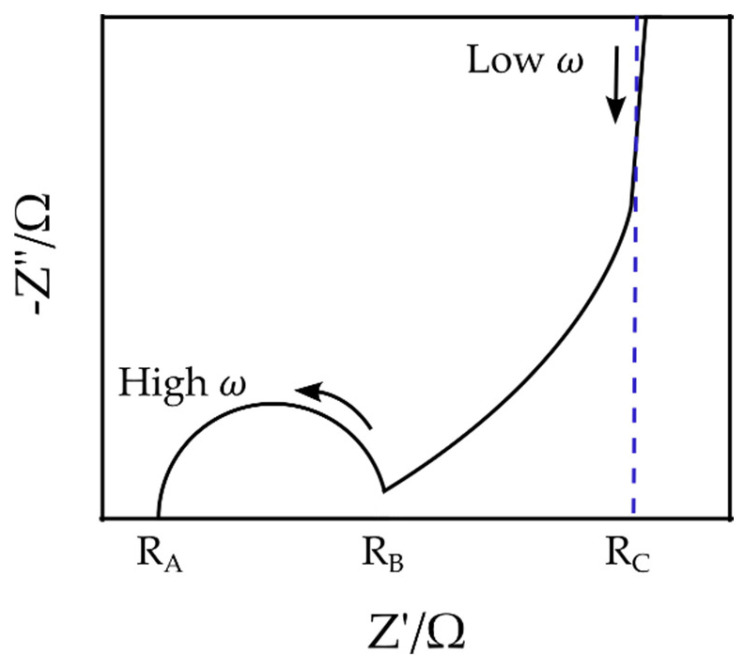
Schematic of a typical Nyquist plot for EDLC supercapacitors.

The Nyquist plot of a typical EDLC supercapacitor generally consists of a semi-circle at high frequencies between points $R_{A}$ and $R_{B}$, a nonvertical line at intermediate frequencies between points $R_{B}$ and $R_{C}$, and a nearly vertical line at low frequencies beyond the point $R_{C}$ [46] (Figure 4). Although EIS can be applied for the characterization and measurement of supercapacitors’ impedance, it can only provide the total impedance, and it is challenging to determine the independent role of each part, e.g., the double-layer and the space charge layer capacitances. Therefore, multiple and often inconsistent physical interpretations of experimental Nyquist plots of EDLCs have been proposed in the literature. The resistance $R_{A}$, for example, has been associated with either the bulk electrolyte resistance [47,48,49,50,51,52,53] or the equivalent series resistance (ESR) [54,55,56,57]. Similarly, there are multiple and often contradicting interpretations for other elements, such as the resistance $R_{B}$ and $R_{C}$ [51,52,58].

In other words, such EC models are not unique, and several models can generate identical impedance results [59], undermining intrinsic correlations of elements with material properties [60]. This has caused independent researchers to use differing EC models to fit the experimental EIS data to explain key physicochemical phenomena involved in an SSC. 

**Figure 5:**
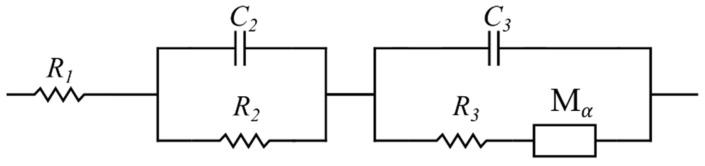
Extended Randles equivalent circuit.

In the conventional mathematical modeling approach, the commonly used impedance data fitting software (ZView, Scribner Associates Inc., NC, USA) was employed to simulate the impedance data. Based on previous working experience and by testing various potential ECs, an extended Randles equivalent circuit showed the best fit to the Nyquist plot and was therefore selected (see Figure 5). Here, element $M_{\alpha }$ is called the modified restricted linear diffusion, and its impedance is given by [61]:

(1) $$Z\left(M_{\alpha }\right)=\frac{R_{d}\coth{\left(\tau_{d}j2\pi \omega \right)}^{\alpha /2}}{{\left(\tau_{d}j2\pi \omega \right)}^{\alpha /2}}$$

where $R_{d}$ is the diffusion resistance, $\tau_{d}$ is the time constant, and α is the dispersion parameter [62]. Nevertheless, it should be acknowledged that Randles circuit models contain physically defined elements. However, this physical relevance did not play a role during the fitting stage. In other words, the circuit model was solely selected because it exhibited the best fitting capability.

### 3.2. Physicochemical Approach to Modeling

In this section, the simulated EIS and the corresponding material properties are introduced. Using physics-based approaches, the impedance model of the electrode is presented, and then a similar methodology is followed for modeling the gel electrolyte impedance.

The energy storage mechanism in EDLCs is explained by the Gouy–Chapman–Stern model [63]. According to this model, energy is stored through the transportation and accumulation of electrolytic ions to a charged interface with a surface potential of ($\psi_{s}$), constructing a double-layered structure of a couple of atomic layers of ions at the electrode interface, called the Stern layer. Adjacent to the Stern layer, a much thicker diffusion layer forms, called the diffusive space charge layer, in which an ionic concentration gradient exists [64]. Among the theoretical models, the Stern–Gouy–Chapman model successfully describes the ionic distribution within the cell. Therefore, it has been widely used by researchers, such as in reference [63].

**Figure 6:**
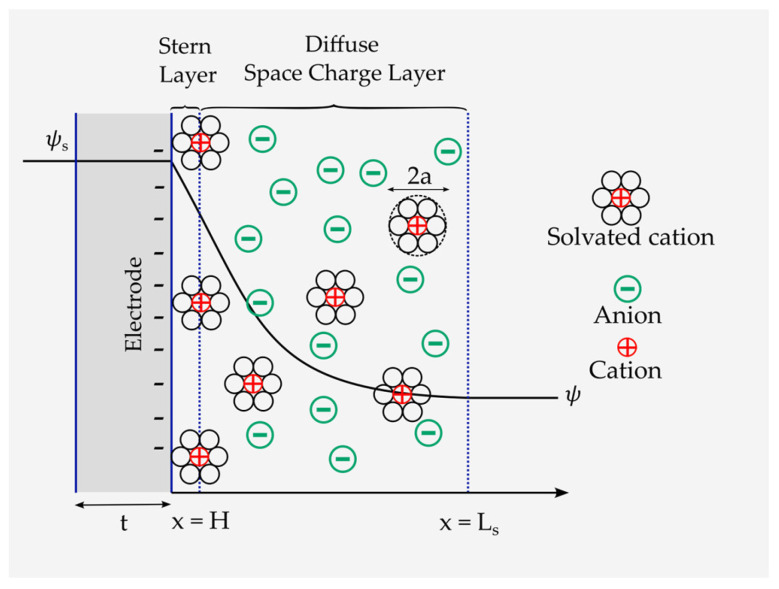
Schematic representation of the Gouy–Chapman–Stern model and the formation of the Stern and space charge layers.

The Stern–Gouy–Chapman model comprises a compact layer and a diffuse space charge layer (Figure 6). In this model, ions are considered to have a finite size and can only approach the electrode surface within the limits of their ionic radii. The Stern layer has a length of H. The diffuse space charge layer extends to the length $L_{s}$. Moreover, the EDL is formed at a negatively charged electrode by diffusion and adsorption of ions of an effective diameter of 2a. In addition, $\psi_{s}$ and ψ are the electrode surface and the electrode/electrolyte interface potentials, respectively. It is also assumed here that the electrolyte thickness $L_{e}$ is infinite, i.e., $L_{s}\ll L_{e}$. Nonetheless, the closest approach of the ion toward the electrode surface also depends on the solvation of the ion in the solvent because, in this case, the radius of the ion increases (see Figure 6). The plane formed by the centers of the solvated ions is the Stern layer. Solvated ions can interact only with the electrode through long-range electrostatic forces and, hence, are called nonspecifically adsorbed ions. The same double layer forms on the positively charged electrode as well (see Figure 3).

#### 3.2.1. Porous Electrode Model

**Figure 7:**
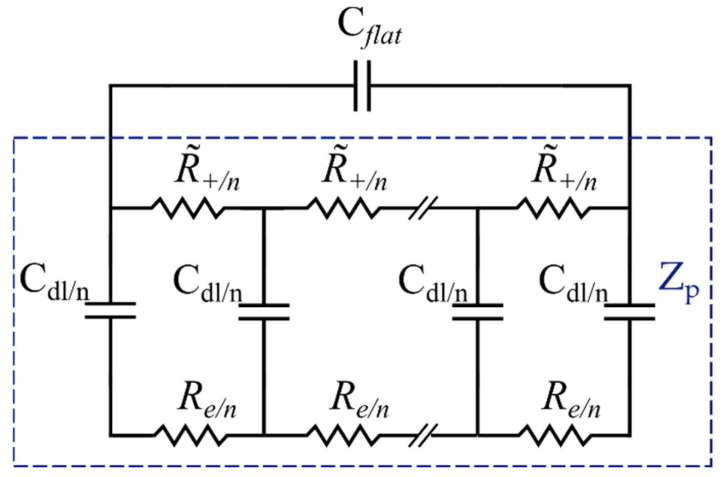
Transmission line model for nonideal porous electrodes.

Porous electrodes are usually used in SSCs due to their high specific surface area [65]. Bisquert et al. developed a model for porous electrodes based on the assumption that the electrode material is not ideally conducting compared to the electrolyte [66,67,68]. To simulate the impedance of the pores in an electrode ($Z_{p}),$ a transmission line with two branches (pathways) is used, as shown in the blue rectangle in Figure 7. This configuration assumes ionic conduction by the solution in the pores and electronic conduction in the solids [35]. The two branches are connected with a capacitor, and the circuit is repeated in n loops, where n is the total number of pores in the system and is defined as $n=L_{e}/Χ$, where Χ is the element thickness. Microporous carbon structures with a narrow micropore size distribution are disadvantageous for electrolyte ion diffusion [69]. However, for the sake of simplicity, in this electrode impedance model, it is assumed that ionic species in the pores can be exchanged freely within the bulk solution. Electrons are also assumed to move freely at the substrate interface [35].

**Figure 8:**
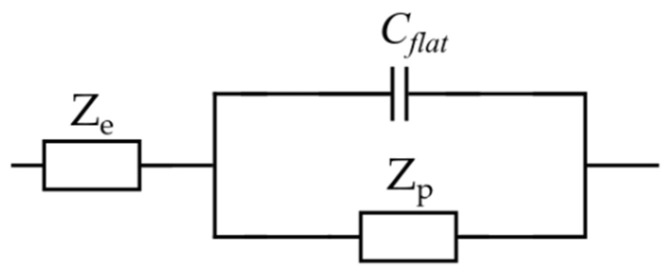
Total porous electrode EC model.

Lasia et al. [70] theorized the contribution of the outer flat layer to the total impedance of the porous electrode by adding an additional capacitor, $C_{\mathrm{flat}}$, to the EC (Figure 8). The flat layer contribution becomes insignificant in a high-frequency range where the penetration length is much shorter than the pore length. However, its effect cannot be ignored at low frequencies. Thereby, in Figure 8, $Z_{e}$ and $Z_{p}$ represent the representative gel-polymer electrolyte impedance and the porous electrode elements, respectively. $C_{\mathrm{flat}}$ refers to the capacitance of the outer layer in the electrode.

Furthermore, based on Lasia’s assumption, the electrolyte solution impedance is only described by a resistor. However, in the case of a gel electrolyte, such an assumption does not portray an accurate description of the electrolyte impedance. Therefore, a representative impedance element, $Z_{e}$, for the electrolyte was considered (Figure 8), which will be discussed in detail in the following section. Each element in the proposed EC model for the electrode will now be defined and associated with the material properties. The total impedance of the porous electrode EC model is given by the following equation [35]:

(2) $$Z_{c}=\frac{1}{j\omega C_{\mathrm{flat}}+1/Z_{p}}.$$

As stated by Bisquert [71], the pore impedance can be modeled using Equations (3) and (4):

(3) $$Z_{p}=\frac{{\tilde{R}}_{+}R_{e}}{{\tilde{R}}_{+}+R_{e}}l\left[1+\frac{2}{\Lambda^{1/2}\sinh\Lambda^{1/2}}\right]+\frac{{\tilde{R}}_{+}^{2}+R_{e}^{2}}{\Lambda^{1/2}\left({\tilde{R}}_{+}+R_{e}\right)}l\coth\Lambda^{1/2},$$

(4) $$\Lambda^{1/2}=l\sqrt{\left({\tilde{R}}_{+}+R_{e}\right)j\omega c_{dl}}$$

where ${\tilde{R}}_{+}$ is the specific ionic transport resistance defined as ${\tilde{R}}_{+}=R_{+}/l$, in which $R_{+}$ and l are the ionic transport resistance in the electrolyte and unit pore length in the electrolyte phase within the electrode pores, respectively. ${\tilde{R}}_{+}$ is derived using the integration of the ionic conductivity of the electrolyte, $\sigma_{e,+}$, over the electrolyte length, $L_{e}$, as expressed by [72]:

(5) $${\tilde{R}}_{+}=\frac{1}{l}\int_{0}^{L_{e}}\frac{dx}{A\sigma_{e,+}}=\frac{RTL_{e}}{{\mathrm{lz}}_{+}^{2}F^{2}AD_{+}c_{+}}$$

where A is the electrode surface area, and $z_{+}$ is the ion valence. Moreover, $c_{+}$ is the initial mobile ions concentration in the electrolyte, and $D_{+}$ is the bulk diffusivity of ions in the electrolyte. R and F are the gas and Faraday constants, respectively. T is the absolute temperature. In Equation (3), $\Lambda^{1/2}$ is a nondimensional parameter representing the inverse of the penetration length. Furthermore, $R_{e}$ is the resistance per unit pore length of the electrode material and can be calculated as the internal resistance per unit pore length of a porous electrode and is given by the below equation. [73]:

(6) $$R_{e}=\frac{R_{\mathrm{int}}}{l}=\frac{1}{l}\frac{L_{e}}{\sigma_{c}+\kappa }\left[1+\frac{2+\left(\frac{\sigma_{c}}{\kappa }+\frac{\kappa }{\sigma_{c}}\right)\cosh\Gamma }{\Gamma \sinh\Gamma }\right]$$

where $R_{\mathrm{int}}$ is the internal resistance of the porous electrode. This quantity combines the kinetic resistance and the ohmic drop in the entire cell. In Equation (6), $\sigma_{c}$ and κ are the conductivity of the electrode matrix material and the electrolyte, respectively. Furthermore, parameter Γ is defined as below [73]:

(7) Γ=θi0αa+αcFLe2RT1σc+1κ12.

Here, θ is called the specific interfacial area, and with the simplified assumption of pores being spherical, it can be defined as $\theta =3\left(1-\chi \right)/r_{p}$, where χ is the porosity volume fraction, and $r_{p}$ is the spherical pore radius [73]. Moreover, $\alpha_{a}$ and $\alpha_{c}$ are the anodic and cathodic transfer coefficients, and the relation $\alpha_{a}+\alpha_{c}=z$ is generally applicable. $i_{0}$ is the exchange current density [43]. Finally, it can be seen here that two key parameters in modeling porous electrodes are pore depth, l, and pore radius, $r_{p}$ [35].

The double-layer capacitance per unit of pore length, $c_{\mathrm{dl}}$, is defined as $c_{\mathrm{dl}}=C_{\mathrm{dl}}/l$. According to the Gouy–Chapman–Stern model, the total electric double-layer capacitance, $C_{\mathrm{dl}}$, consists of the Stern layer and the diffuse layer capacitances in series. Thus, the differential capacitance is given by [74]:

(8) $$\frac{1}{C_{\mathrm{dl}}}=\frac{1}{C_{\mathrm{stern}}}+\frac{1}{C_{\;}{}_{\mathrm{diffuse}}}.$$

In the case of planar electrodes with binary and symmetric electrolytes, the capacitance of the Stern layer depends on the electric field and the length of the Stern layer, which is defined as [75]:

(9) $$C_{\mathrm{stern}}=\frac{\varepsilon_{0}\varepsilon_{r}}{H}$$

where $\varepsilon_{0}$ is the permittivity of space and $\varepsilon_{r}$ is the relative permittivity of the Stern layer, which is a function of the electric field. Here, $\varepsilon_{r}$ is assumed to be constant, $\varepsilon_{r}\left(E\right)=\varepsilon_{r}\left(0\right)$, since $E<10^{7}\;Vm^{-1}$ [76,77,78]. In addition, H is the length of the Stern layer, and H=a, where a is the effective ion radius [79]. 

Employing the modified form of the Poisson-Nernst-Planck equations (MPNP) for planar electrodes, the diffuse layer specific capacitance is given by [74]:

(10) $$C_{\mathrm{diffuse}}=\frac{\frac{\varepsilon }{\lambda_{D}}\sinh\left(\frac{ze\psi_{D}}{2k_{B}T}\right)}{\left[1+2\nu \sin h^{2}\left(\frac{ze\psi_{D}}{2k_{B}T}\right)\right]\sqrt{\frac{2}{\nu }\ln\left[1+2\nu \sin h^{2}\left(\frac{ze\psi_{D}}{2k_{B}T}\right)\right]}}$$

where $\psi_{D}$ is the voltage drop across the double layer, $\nu =2a^{3}C_{0}$ is a dimensionless measure of nondiluteness, and e is the electron charge. Additionally, $k_{B}$ is the Boltzmann constant and $\lambda_{D}$ is the screening length defined by [74]:

(11) $$\lambda_{D}=\sqrt{\frac{\varepsilon kT}{2z^{2}e^{2}c_{0}}}.$$

As Equation (10) suggests, $C_{\mathrm{diffuse}}$ is evidently not a constant value and is a function of the potential drop in the diffuse layer. In other words, $C_{\mathrm{diffuse}}$ becomes infinitesimal at small values of $\left(ze\psi_{D}/2k_{B}T\right)$ and reaches the maximum in intermediate values and finally approaches zero at higher values [74]. However, it can be assumed that the diffuse capacitance is constant at $\psi_{D}=10K_{B}T/ze$, as suggested in [74]. For a planar spacing of *L*, $C_{\mathrm{flat}}$ in Equation (2) can be conveniently calculated as below [43]:

(12) $$C_{\mathrm{flat}}=\frac{\varepsilon_{0}\varepsilon_{i}A}{L}.$$

#### 3.2.2. Gel-Polymer Electrolyte Model

**Figure 9:**
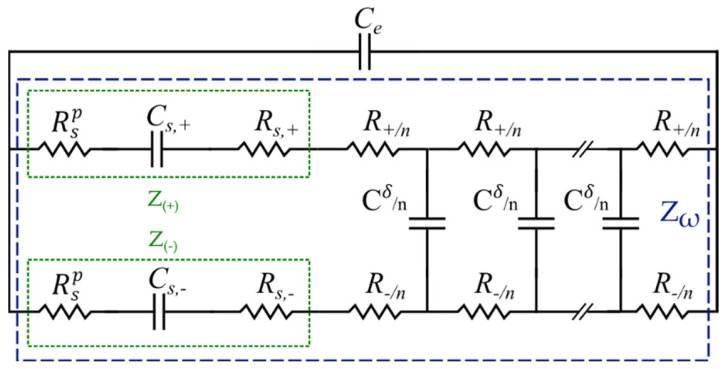
EC model for gel-polymer electrolyte as developed in [44].

Accurate modeling of a gel-polymer electrolyte, on the other hand, requires a more sophisticated description of the involved processes and key factors, which are relatively inapplicable in their liquid counterparts [80]. One of the most critical factors is the effect of the space charge layer in the gel-polymer electrolytes [44]. To investigate the intrinsic relationships, electrochemical models have to be implemented again to obtain the charge and the electrostatic potential distribution inside the SSC, particularly in the space charge layer [33]. Several investigations view the space charge layer as an ideal capacitor [64,81]. However, such an assumption does not correlate well with the experimental observations at low frequencies [82]. To tackle this problem and accurately quantify the space charge layer impedance, the MPNP relationships [83] have been applied by Liu et al. [44] to derive a well-founded EC model, as shown in Figure 9. In this model, the space charge layer polarization impedance was introduced to entail the frequency dependence of the charge density and the interfacial impedance [84,85,86]. Furthermore, all of the elements are quantified and assigned to the gel-polymer electrolyte properties. The proposed EC consists of a transmission line parallel to the electrolyte bulk capacitance, $C_{e}$. Note that throughout the text, the + and − notations represent the contribution of ions and electrons, respectively, and the subscript i  represents either + or −. In Figure 9, $C_{s,+}$ and $C_{s,-}$ represent the space charge layer capacitances, whereas $R_{s,+}$ and $R_{s,-}$ are the space charge layer resistances. Moreover, $R_{s}^{p}$ symbolizes the polarization resistance, while $Z\left(+\right)$ and $Z\left(-\right)$ show the space charge layer impedance due to the ions and electrons, respectively. Additionally, $C^{\delta }$ is the chemical capacitance and $Z\left(\omega \right)$ marks the total transmission line impedance. It should be noted that electrons move with a relatively low velocity in the gel-polymer electrolyte at room temperature [44]. However, their contribution to impedance in gel electrolyte cannot be totally ignored. Therefore, a separate pathway for electron flow resistance in the solid electrolyte was also considered here to derive a comprehensive model, which is well suited for the entire frequency regime.

In the EC model shown in Figure 9, $R_{i}$ and $C_{i}$ refer to the electrolyte bulk resistance and dielectric capacitance contributed by the ions and electrons, respectively, and *n* is the number of transmission line elements (compare Figure 7) [87]. The charge accumulation at the electrode/electrolyte interface results in the space charge layer impedance $\;Z\left(i\right)$, and as a consequence, the impedance is proportional to the charge density. In this study, in order to focus on the interface layer, only the space charge layer impedance of the half-cell (the cathode) is considered, while the anode space charge layer impedance is disregarded. This simplification corresponds to the case of an infinite gel-polymer electrolyte thickness and is reasonable when the space charge layer is much smaller than the electrolyte $L_{s}\ll L_{e}$, which is satisfied in the current study. In several studies, $Z\left(i\right)$ is either considered as a pure capacitor or represented as the space charge layer capacitance $C_{s,i}$ in parallel with the space charge layer resistance $R_{s,i}$ [88]. The space charge layer impedance $Z\left(i\right)\;$ is, however, a function of the charge density; thus, the capacitor $C_{s,i}$ and the resistance $R_{s,i}$ are frequency dependent. Although numerically solvable, the results fail to correlate with material properties; thus, researchers usually calculate the values at the equilibrium state, i.e., ω=0. This ignores the polarization effect and frequency perturbation. Hence, Liu et al. introduced the polarization resistance to solve this problem. The space charge layer impedance $Z\left(i\right)\;$ in this model consists not only of the space charge layer capacitance $C_{s,i}$ and the space charge layer resistance $R_{s,i}$ but also of an innovatively defined space charge layer polarization resistance, $R_{s}^{p}$ (see Figure 9). This yields the impedance $Z\left(i\right)$ [44]:

(13) $$Z\left(i\right)={\left(j\omega C_{s,i}\right)}^{-1}+R_{s,i}+R_{s}^{p}.$$

The concentration distribution according to the MPNP formulations is defined by the modified Boltzmann distribution [74]:

(14) ci=ci,∞e−ziFΦRT1+c˜i,∞e−ziFΦRT−1

where ${\tilde{c}}_{i,\infty }$ is the dimensionless concentration [74]. The Poisson equation is given in the following, with ρ being the charge of the immobile species $\rho =c_{+}-c_{-}$, $z_{i}$ the valence, and $z=\left|z_{i}\right|$ [44]:

(15) $$\nabla^{2}\Phi =-\frac{F}{\varepsilon_{0}\varepsilon_{i}}\left(z_{+}c_{+}+z_{-}c_{-}-z\rho \right)$$

The analytical solution of Equation (15) gives the electric field E [89]:

(16) E=2RTcmaxε0εiln1+c˜+,∞e−zFΦRT−1+ln1+c˜−,∞ezFΦRT−1+2FΦρ˜cmaxε0εi.

In Equation (16), $c_{\max}$ is the maximum charge concentration in the electrolyte, and $\tilde{\rho }$ is the dimensionless ρ. The space charge layer capacitance $C_{s}$ is then analytically calculated from partial derivate of the surface charge density Q with respect to the electrostatic potential Φ [44]:

(17) $$C_{s}=-\frac{\partial Q}{\partial \Phi }=-\frac{\partial \left(-AE\varepsilon_{0}\varepsilon_{i}\right)}{\partial \Phi }=A\varepsilon_{0}\varepsilon_{i}\frac{\partial E}{\partial \Phi }.$$

In the investigated SSC, ${\tilde{c}}_{+}={\tilde{c}}_{-}=0.5$ and $\tilde{\rho }=0$. In addition, to simplify the electrolyte model, it is assumed that the KCl ions do not contribute to ion conductivity due to their low concentration. Therefore, $C_{s,+}=C_{s,-}$, which is analytically expressed by [74]:

(18) Cs,i=AFcmax−zc˜i,∞e−zFΦRT1+c˜i,∞e−zFΦRT−1+Fc˜+cmax2RTcmaxε0εiln1+c˜i,∞e−zFΦRT−1+2FΦc˜icmaxε0εi.

In the EC model for the gel electrolyte, $R_{i}$ represents the resistance of either electrons or ions in the electrolyte, which can be calculated using the conductivity $\sigma_{i}$ given by the below relations [72]:

(19) $$\sigma_{i}=\frac{z_{i}^{2}F^{2}D_{i}c_{i}}{RT},$$

(20) $$R_{i}=\int_{0}^{L_{e}}\frac{dx}{A\sigma_{i}}=\frac{RTL_{e}}{z_{i}^{2}F^{2}AD_{i}c_{i}}.$$

The space charge layer resistance $R_{s,i}$, originated by the charge accumulation or depletion at the space charge layer, is merely caused by the deviation from the bulk contribution and can be obtained by the following [90]:

(21) $$R_{s,i}=\frac{RT}{z_{i}^{2}F^{2}AD_{i}}\int_{0}^{\lambda_{s}}\frac{dx}{{\tilde{c}}_{i}}$$

where $\lambda_{s}$ and $D_{i}$ are the length of the space charge layer and the bulk diffusivity, respectively. As previously mentioned, the $R_{s,i}\;$ are calculated at an equilibrium state (*ω* = 0). Nevertheless, the charge density in the space charge layer is a function of the frequency. Therefore, $C_{s}$ and $R_{s,i}$ are not independent of frequencies. To contain this dependence, the space charge layer polarization impedance $R_{s}^{p}$ was introduced by Liu et al. [44]. The polarization resistance is a function of the electrolyte resistance $R_{i}\;$ and the nondimensional frequency $\tilde{\omega }=\omega /\omega_{c}$ with $\omega_{c}$ = 1 Hz. For an ideally blocking electrode at the interface, $R_{s}^{p}$ is given by [44]:

(22) $$R_{s}^{p}=\frac{R_{+}R_{-}}{R_{+}+R_{-}}\frac{1}{\tilde{\omega }}.$$

According to the EC model for gel-polymer electrolyte (Figure 9), the total space charge layer impedance is as follows:

(23) $$Z\left(+\right)=Z\left(R_{s}^{p}\right)+Z\left(C_{s,+}\right)+Z\left(R_{s,+}\right)=R_{s}^{p}+\frac{1}{j\omega C_{s,+}}+R_{s,+},$$

(24) $$Z\left(+\right)=Z\left(R_{s}^{p}\right)+Z\left(C_{s,-}\right)+Z\left(R_{s,-}\right)=R_{s}^{p}+\frac{1}{j\omega C_{s,-}}+R_{s,-}.$$

Moreover, $C^{\delta }$ represents the electrolyte chemical capacitance, and as noted by Jamnik and Maier [87], it is directly related to ambipolar diffusion in mixed conducting materials. $C^{\delta }$ is defined as the second derivative of the Gibbs free energy by the changes of the component chemical potential $\mu_{i}$ due to the concentration $c_{i}$ variations [91]. This capacitance is related to the material volume and can be comparatively larger compared to the electrolyte bulk capacitance $C_{i}$. However, the chemical capacitance $C^{\delta }$ line is omitted at high frequencies. Nevertheless, it cannot be disregarded at low frequencies. As the electrolyte remains locally electroneutral in the homogenous region, the ionic and electronic chemical capacitors are in serial connection. Therefore, the total chemical capacitance $C^{\delta }$ is given by [90]:

(25) $$C^{\delta }=\frac{c_{\max}{\left(Fz\right)}^{2}AL}{RT}\left[\frac{1}{{\tilde{c}}_{+}\left(1-{\tilde{c}}_{+}\right)}+\frac{1}{{\tilde{c}}_{-}\left(1-{\tilde{c}}_{-}\right)}\right]$$

Having defined the above elements, the transmission line impedance $Z_{w}$ can now be obtained (blue box in Figure 9):

(26) $$Z_{w}=\frac{R_{+}R_{-}}{R_{+}+R_{-}}+\frac{\left[R_{+}^{2}Z_{-}+R_{-}^{2}Z_{+}\right]\tanh\left[\sqrt{j\omega C^{\delta }\left(R_{+}+R_{-}\right)}\right]+Z_{-}Z_{+}\left(R_{+}+R_{-}\right)\sqrt{j\omega C^{\delta }\left(R_{+}+R_{-}\right)}}{\left(R_{+}R_{-}\right)\left[\left(R_{+}+R_{-}\right)\tanh\left[\sqrt{j\omega C^{\delta }\left(R_{+}+R_{-}\right)}\right]+\sqrt{j\omega C^{\delta }\left(R_{+}+R_{-}\right)}\left[Z_{+}+Z_{-}\right]\right]}$$

Finally, the total gel-polymer electrolyte impedance $Z_{e}$ is given as follows:

(27) $$Z_{e}=\frac{1}{j\omega C_{e}+1/Z_{w}}$$

In which the electrolyte capacitance is calculated by [73]:

(28) $$C_{e}=\frac{\varepsilon_{0}\varepsilon_{i}A}{L_{e}}$$

### 3.3. Total Physicochemical Model

**Figure 10:**
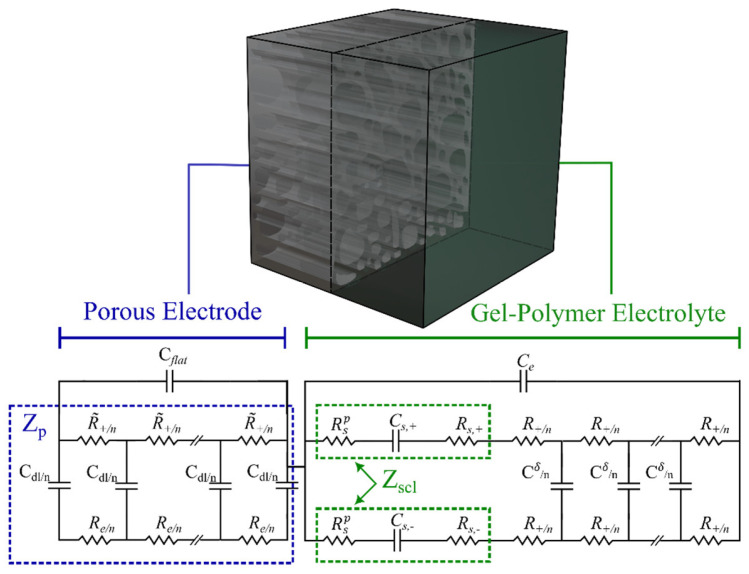
Proposed model for an SSC including a porous electrode and solid electrolyte.

Figure 10 shows the half-cell of a generic SSC with a porous electrode and a gel-polymer electrolyte next to the overall proposed EC model, developed previously using the physicochemical approach. As mentioned earlier, this model is based on the finite electrolyte assumption ($L_{s}\ll L_{e}$). Note that a similar impedance analysis can be applied to the right-hand electrolyte interface if included. The space charge layer impedance $Z_{\mathrm{scl}}$ is presented in the green box within the gel-polymer electrolyte EC (Figure 10).

The total impedance of the total SSC, $Z_{\mathrm{SSC}}$, is the summation of the porous electrode (cathode) impedance, $Z_{c}$, derived by Equation (2) and the impedance of the gel-polymer electrolyte, $Z_{e}$, as given Equation (27):

(29) $$Z_{\mathrm{SSC}}=Z_{c}+Z_{e}$$

The physicochemical model in Equation (29) was used to fit the experimental EIS data of the SSC. Furthermore, the values of the elements in the proposed equivalent circuit model were calculated analytically and then compared to the same parameters acquired using fitting of the experimental data. All of the parameters used in the impedance simulation were extracted either from the characterization of the developed SSC or from the literature (see Table A1). The values of the circuit elements in the proposed EC model for the porous electrode were determined using Equations (5)–(12), while the elements’ values in the gel-electrolyte’s EC were acquired using Equations (17)–(22) (compare Table 2).

### 3.4. Fitting of EIS Data

The aim of data fitting is to determine the EC model’s parameters $\vec{p}$ (see Table 2). In order to fit the EIS data, an interior point technique (compare, for example, [92]) and a global optimization approach according to Ugray et al. [93] were used to find the global minimum of constrained nonlinear multivariable function of the impedance model. Using a multistart heuristic algorithm, the local solver runs repeatedly to locate a solution with the lowest value of Equation (30). The programming was carried out by the numeric computing platform (MATLAB 9.12, MathWorks, Inc., Natick, MA, USA). For this fitting approach, a weighted sum of squares [35]:

(30) $$h\left(\vec{p}\right)∶=\overset{N}{\underset{k=1}{\sum }}\left\{w_{k}^{\prime }{\left[Z_{k}^{\prime }-Z_{k,\mathrm{calc}}^{\prime }\right]}^{2}+w_{k}^{\prime \prime }{\left[Z_{k}^{\prime \prime }-Z_{k,\mathrm{calc}}^{\prime \prime }\right]}^{2}\right\}$$

Of the differences between the experimentally determined impedances, $Z_{k}^{\prime }$ and $Z_{k}^{\prime \prime }$, and the model’s impedance, $Z_{k,\mathrm{calc}}^{\prime }$ and $Z_{k,\;\mathrm{calc}}^{\prime \prime }$ was minimized by choosing the best values of the adjustable parameters $\vec{p}$. Here, N is the number of all measured frequencies. In the current study, a modulus weighting $w_{k}^{\prime }=w_{k}^{\prime \prime }=1/{\left|Z_{k}\right|}^{2}$ of Equation (30) was realized.

As a measure of the relative error of the approximated impedances, the following equation was used:

(31) $$r^{2}=\frac{1}{2N}\sum_{k=1}^{N}\left\{{\left[\frac{Z_{k}^{\prime }-Z_{k,\mathrm{calc}}^{\prime }}{Z_{k}^{\prime }}\right]}^{2}+{\left[\frac{Z_{k}^{\prime }-Z_{k,\mathrm{calc}}^{\prime \prime }}{Z_{k}^{\prime \prime }}\right]}^{2}\right\}$$

Using this error measurement in Equation (31), the approximation ability was assessed for the different used EC models.

## 4. Results and Discussion

### 4.1. Microscale Analysis of Electrode Surface Morphology

**Figure 11:**
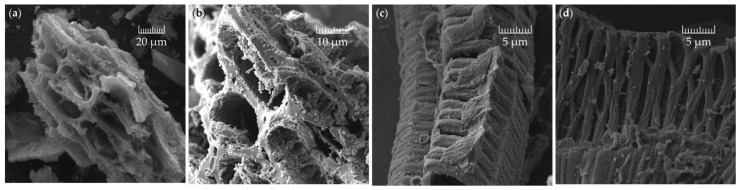
FE-SEM imaging of (**a**) porous bc−GP electrode; (**b**) under higher magnification; (**c**,**d**) same sample from a different view and under higher resolution.

The field emission scanning electron microscopy (FE-SEM) images of the electrode material made from bc−GP reveal the surface morphology of bc−GP carbon (Figure 11). The surface of the activated carbon exhibits the characteristics of a macro porous structure which extends to the surface of the activated carbon and mainly presents a circle or ellipse shape and forms a rib-like structure throughout the electrode (see Figure 11c,d). This circular and elliptical macro pore structure could result in elevated ion transport efficiency, which reduces the resistance in the process of electrolyte ion transport [94]. Furthermore, the structure of the electrode is uniformly porous.

### 4.2. Comparison of Developed Models and Experimental Data

**Figure 12:**
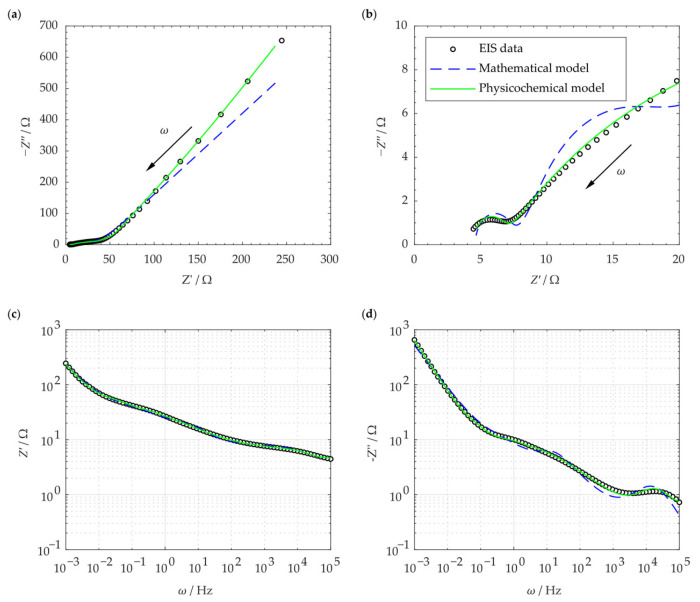
Nyquist plots of the SSC and its modeling as shown for (**a**) the entire frequency spectrum; (**b**) the high-frequency range; (**c**) the real impedance $Z^{\prime }$ gainst frequency ω; (**d**) the imaginary impedance Z″ against frequency ω.

Figure 12a shows the result of the electrochemical impedance spectroscopy for the SCC cell. Each data point in the curve corresponds to a specific frequency. The green line represents the complex impedance generated by the proposed physicochemical model as calculated in Equations (2), (27), and (29). The blue line shows the complex impedance given by the commonly used mathematical model as obtained by the extended Randles circuit model shown in Figure 5. Figure 12b shows the same plot, magnified at the high-frequency section. The proposed physicochemical model fits the EIS data well throughout the entire frequency range. The mathematical model, however, shows a slight difference at lower frequency range along with a notable deviation at the higher frequency section. Figure 12c,d show the real and imaginary impedance as a function of the frequency, respectively. The plot and the models are well-fitted, and the physicochemical EC model indicates a more accurate approximation.

Figure 12a is the Nyquist plot of the SSC and is similar to the typical EDLC supercapacitors’ impedance curve (compare Figure 4). In order to compare the capability of the models in simulating the SSC’s EIS data, both mathematical and physicochemical models were independently used to approximate the experimental data. Using Equation (31), the relative error of the approximated impedances in the mathematical and physicochemical models was calculated to be 15.1% and 3.7%, respectively. It should be noted that the even though the mathematical approach to modeling can fit the EIS data relatively well, its application should be reconsidered for a number of reasons. First of all, in the extended Randles circuit adopted here (see Figure 5), the nonintuitive circuit elements, such as $M_{\alpha }$, do not correlate clearly with the material properties (see Equation (1)). Furthermore, this approach cannot explain the high impedance at the space charge layer at low frequencies. Finally, such models are not unique and various modeling efforts can generate different EC models. On the other hand, the proposed physicochemical model in the present study shows a lower relative error in approximation and includes the effect of the interfacial impedance and its dependence on frequency. Moreover, its circuit elements are in a well-defined correlation with the physical phenomena and material properties. 

The reason for the better approximation ability of the physicochemical model can be attributed to the following strengths of this model. First of all, the important role of space charge layer impedance and its association with frequency is not disregarded in this model. In the proposed physicochemical circuit model, the space charge layer impedance contains a polarization impedance, $R_{s}^{p}$, which is frequency dependent (see Equation (22)). In fact, $R_{s}^{p}$, which is caused by the perturbed charge density within the space charge layer, plays an important role at low frequencies that cannot be ignored. Similarly, other key circuit elements, such as chemical capacitance, $C^{\delta },$ are included, which help the model to be comprehensive. Finally, the number of parameters (i.e., the elements in the corresponding EC) are higher compared to the mathematical one. This helps maintain a low relative error in the fitting process. In contrast, general guidelines in mathematical circuit modeling recommend to minimize the number of circuit elements [35]. Overall, the fitting results in Figure 12 indicate the relevance and suitability of our proposed circuit model in analyzing the EIS of SCCs. 

Using the corresponding formulations discussed in previous sections, the parameters in the elements of the physicochemical EC model were calculated analytically. The constants used in the analytical investigation were mostly obtained from values in the literature, as listed in Table A1, whereas the others were experimentally measured. Both the analytical values and the values obtained from the best fits are listed in Table 2. However, a comparison of these parameters reveals poor compliance. This can be attributed to the error in value estimations or simplifications and assumptions made in the analytical analysis. The set of parameters with the lowest error was obtained by choosing a global optimization algorithm. This resulted in a good approximation of the EIS data using the physicochemical model. Nevertheless, more experimental and numerical analysis are required to investigate the root cause in the different results obtained from the physicochemical model and the analytical estimations.

## 5. Conclusions

A physicochemical modeling approach was used to build an equivalent circuit model to approximate the impedance response of a solid-state supercapacitor prepared from biomass and gel polymer. All circuit elements were quantified and associated with physicochemical properties within this framework. Compared to a conventionally used mathematical model, the physically meaningful proposed model can accurately produce the supercapacitor’s impedance data. In addition, one of the unique aspects of the proposed physicochemical model was that the material properties concretely defined each circuit element and that the electrode/electrolyte impedance was clearly quantified. Moreover, due to its excellent approximation capability, this physicochemical model can be applied to model the impedance data in various solid-state supercapacitors. Nevertheless, further research is required to compare the supercapacitor’s properties and the model parameter. Finally, the physicochemical model developed here can be used to investigate the electrochemical mechanisms in structural supercapacitors with gel-polymer electrolytes, which can eventually result in higher efficiencies for practical applications.

## Figures and Tables

**Table 1:** Pore morphology measurement results for the bc−GP electrode.

Parameter	Unit	Value	Description
$V_{p}$	${\mathrm{cc}\;g}^{-1}$	$1.540\times 10^{-1}$	Pore volume
$A_{p}$	$m^{2}\;g^{-1}$	$2.689\times 10^{2}$	Surface area
$d_{p}$	m	$1.1031\times 10^{-11}$	Pore width

**Table 2:** Analytically obtained EC and physicochemical model’s parameters.

Parameter $\vec{p}$	Unit	Analytical Value	Value of Best Fit
*Electrode*			
${\tilde{R}}_{+}$	Ω m^−1^	$2.961\times 10^{6}\;$	$6.890\times 10^{-2}$
$R_{e}$	Ω m^−1^	15.32	5.49
l	m	$5\times 10^{-4}$	24.20
$C_{\mathrm{dl}}$	F m^−1^	4.060	0.160
$C_{\mathrm{flat}}$	F	$5.790\times 10^{-11}$	$2.91\times 10^{-2}$
*Electrolyte*			
$C_{e}$	F	$5.790\times 10^{-11}$	$2.10\times 10^{-7}$
$C_{s,+}$	F	$7.780\times 10^{-1}$	$3.35\times 10^{-6}$
$C_{s,-}$	F	$7.780\times 10^{-1}$	2.39
$R_{s,+}$	Ω	$4.250\times 10^{-11}$	14.30
$R_{s,-}$	Ω	$3.330\times 10^{-7}$	6.81
$R_{+}$	Ω	$1.458\times 10^{3}$	$1.58\times 10^{-1}$
$R_{-}$	Ω	$1.434\times 10^{7}$	8.45
$C^{\delta }$	F	$1.813\times 10^{3}\;$	$7.15\times 10^{-3}$

**Figure A1:**
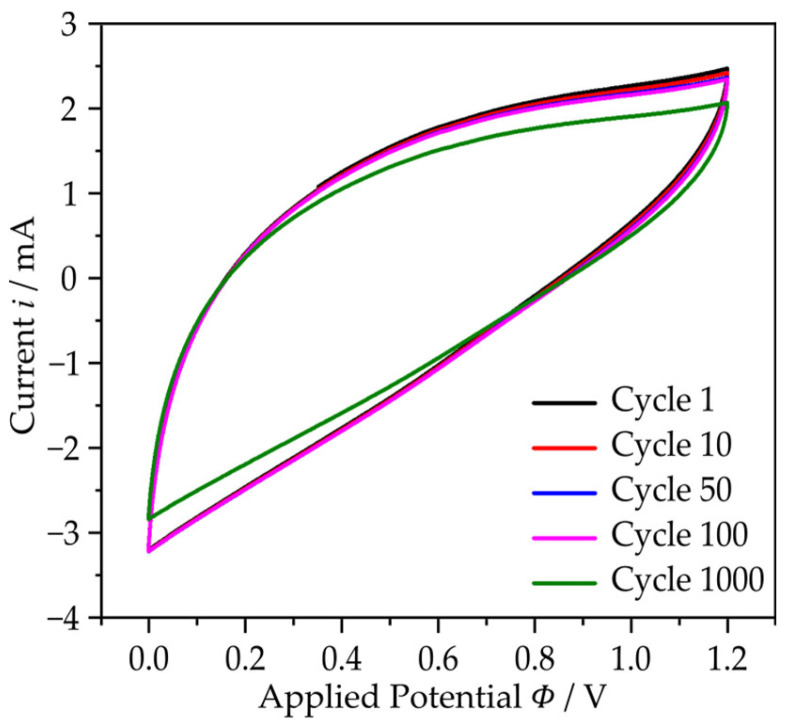
Cyclic voltammogram of the manufactured symmetric SSC in aqueous KOH and gel polymer electrolyte at a current density of 50 mA/g for 1 to 1000 cycles.

## Data Availability

The data presented in this study are available upon request from the corresponding author.

## Appendix A

### Appendix A.1. Cyclic Voltammogram

Cyclic voltammogram (CV) of the developed SSC was conducted in the potential window of 0.0 to 1.2 V at the scan rates from 20 to 200 mV/s. It can be seen that the SSC is extremely stable at high charge–discharge cycles (Figure A1).

### Appendix A.2. Porous Electrode and Gel-Polymer Electrolyte Parameters

In the following table, the parameters of the porous electrode and the electrolyte are listed.

**Table A1:** Gel-polymer electrolyte and porous electrode parameters.

Parameter	Unit	Value	Description	Reference
$L_{e}$ *	m	$3.60\times 10^{-4}$	thickness of the electrolyte	
A *	$m^{2}$	$7.85\times 10^{-5}$	surface area of the electrode plate	
Χ *	m	$7.00\times 10^{-4}$	full element thickness	
t *	m	$1.7\times 10^{-4}$	thickness of the electrode	
l *	m	$5\times 10^{-4}$	total electrode pore depth	
χ *	-	$3.5\times 10^{-1}$	electrode porosity volume fraction	
a	m	$3.00\times 10^{-10}$	effective ionic radius	
$r_{p}$ *	m	$5.51\times 10^{-10}$	spherical pore radius	
$D_{+}$	$m^{2}\;s^{-1}$	$7.84\times 10^{-13}$	diffusivity of Potassium ions in the electrolyte	[95]
$D_{-}$	$m^{2}\;s^{-1}$	$10^{-16}$	diffusivity of electrons in the electrolyte	[96]
$\varepsilon_{0}$	${\mathrm{F m}}^{-1}$	$8.85\times 10^{-12}$	vacuum permittivity	[97]
$\varepsilon_{i}$	-	30	relative permittivity	[98]
$\varepsilon_{r}$	${\mathrm{F m}}^{-1}$	$78.5\times 10^{-12}$	permittivity at the stern/diffuse layer interface	[99]
$E_{a}$	eV	2.5	activation energy	[95]
$\sigma_{e}$	${\mathrm{S m}}^{-1}$	$4.7\times 10^{-2}$	ionic conductivity at 298.15 k in electrolyte	[100]
$c_{\max}$ *	${\mathrm{mol m}}^{-3}$	$2.1\times 10^{3}$	maximum concentration	
$c_{+}$ *	${\mathrm{mol m}}^{-3}$	$1.05\times 10^{3}$	initial mobile ions concentration in the electrolyte	
$c_{-}$ *	${\mathrm{mol m}}^{-3}$	$1.05\times 10^{3}$	initial mobile electron concentration in the electrolyte	
$z_{+}$	-	+1	ion valence	
$z_{-}$	-	−1	electron valence	
$\sigma_{c}$	${\mathrm{S m}}^{-1}$	$4.7\times 10^{-2}$	conductivity of the electrode matrix	[101]
κ	${\mathrm{S m}}^{-1}$	$6.0\times 10^{-5}$	conductivity of the electrolyte	[100]
$i_{0}$*	$A\;m^{-2}$	$6.37\times 10^{2}$	current density	
T *	K	293.15	ambient temperature	
F	${\mathrm{C mol}}^{-1}$	96,485	Faraday constant	[102]
R	${\mathrm{J mol}}^{-1}\;K^{-1}$	8.314	gas constant	[102]
$K_{B}$	${\mathrm{J K}}^{-1}$	$1.380\times 10^{-23}$	Boltzmann constant	[103]

* These parameters were measured for the investigated SSC.
